# Dissociating the relationship between time perception and decisions to delay gratification

**DOI:** 10.3758/s13423-026-02992-7

**Published:** 2026-08-19

**Authors:** Isaac J. George, Morgan G. Moll, Ryan K. Read, Hannah M. Stealey, Thomas W. Elston

**Affiliations:** Department of Neuroscience, University of Texas at Austin, Austin, TX 78712 USA

**Keywords:** Decision making, Time perception, Delay discounting, Delayed gratification

## Abstract

The ability to forgo an immediate reward in favor of a larger, later one is central to healthy decision-making–—yet some people struggle to delay gratification. A prominent theory proposes that people with faster internal clocks experience delays as subjectively longer and more costly, leading them to discount future rewards more steeply. Here, we test this theory’s central prediction by asking whether individual differences in time perception can explain individual differences in temporal decision-making. We recruited 226 participants to complete tasks measuring time perception and delay sensitivity across experiential and hypothetical decisions. We found no relationship between time perception and temporal decision-making. We also found that survey and task-based measures of temporal discounting are unrelated and that neither measure predicts real-world timing-related behaviors. Our findings indicate that time perception and temporal decision-making derive from dissociable cognitive processes and that standard laboratory measures of discounting have limited ecological validity. We suggest that foraging paradigms, which embed time–reward trade-offs in naturalistic behavioral contexts offer a promising path toward more ecologically valid and mechanistically tractable approaches to understanding how the brain trades off time and reward during decision-making.

## Introduction

We constantly face decisions that require weighing immediate rewards against future benefits—for example, choosing whether to wait for a table at a favorite restaurant versus settling for an open table at a less appealing restaurant. This ability to forgo a quick, small reward in favor of a larger, later one is central to healthy decision-making (Bickel et al., 2019; MacKillop et al., 2011; Patros et al., 2016; Petry, 2001a). Critically, these decisions depend not only on the objective duration of delays, but on how long those delays subjectively feel. Consider an impatient diner waiting for that restaurant table—one whose internal clock runs fast may experience the same 20-min wait as subjectively longer and more aversive, making them more likely to leave. This suggests that individual differences in time perception—how quickly or slowly time seems to pass—may be a fundamental mechanism underlying why some people consistently choose immediate gratification while others demonstrate patience for future rewards. Indeed, research suggests that people who struggle to delay gratification have internal clocks that run faster than people who are willing to wait for larger rewards (Moreira et al., 2025; Petry, 2001b; Wittmann & Paulus, 2008; Wittmann et al., 2011). This has led to an “internal clock speed hypothesis” (Wittmann & Paulus, 2008) that, because these individuals experience time as passing more quickly, delays feel subjectively longer and more costly, leading them to discount the value of future rewards more steeply.

This “internal clock speed hypothesis” (Wittmann & Paulus, 2008) has mainly been explored using hypothetical choice scenarios. Researchers typically ask participants to either imagine trade-offs between smaller, sooner and larger, later rewards, or to abstractly report how they perceive different time intervals. For example, Kim and Zauberman (2009) had participants report their subjective perception of future time horizons (3 to 36 months) by adjusting line lengths on a computer screen. They then tested whether these subjective time perceptions (line lengths) predicted the delayed compensation participants would require to forgo an immediate hypothetical $75 payout. Baumann and Odum (2012) took a behavioral approach to measuring time perception—having participants judge whether experienced intervals were “long” or “short”—but still relied on hypothetical monetary choices to assess delay tolerance (e.g., “$50 now or $100 in 6 years?”). Both studies found that people with faster internal clocks were less willing to wait for delayed rewards, providing empirical support for the “internal clock speed hypothesis.”

While these studies suggest that people with faster internal clocks are less patient with imagined delays, most real-world decisions require experiencing, rather than imagining, the passage of time. For example, Patt et al. (2021) compared choice patterns across experiential and hypothetical (survey-based) delay discounting tasks. Like others (Melanko et al., 2009; Reynolds et al., 2006; Smits et al., 2013), the authors found no correlation between people’s decisions in survey-based assessments of temporal discounting and their behavior in experiential discounting. Similarly, Steele et al. (2019) examined the differential influence of hypothetical versus experienced delays and rewards on discounting behavior. The authors reported that experiencing the reward—in their case, receiving real M&M candies versus being told about getting hypothetical M&Ms—had a negligible effect on discounting behavior. However, the authors found that experiencing the delays associated with choices had a large impact on discounting behavior. This description–experience gap (Elston et al., 2021; Hertwig & Erev, 2009; Hertwig & Ortmann, 2001; Navarick, 2004; Reynolds & Schiffbauer, 2004) in the discounting literature raises the question of whether the relationship between time perception and willingness to delay gratification holds when people experience the consequences of their choices. More fundamentally, we ask whether individual differences in temporal processing constrain the ability to delay gratification—do time perception and temporal discounting arise from a single, shared cognitive/neural mechanism or do they operate independently?

## Results

### Online behavioral and psychometric testing

**Fig. 1:**
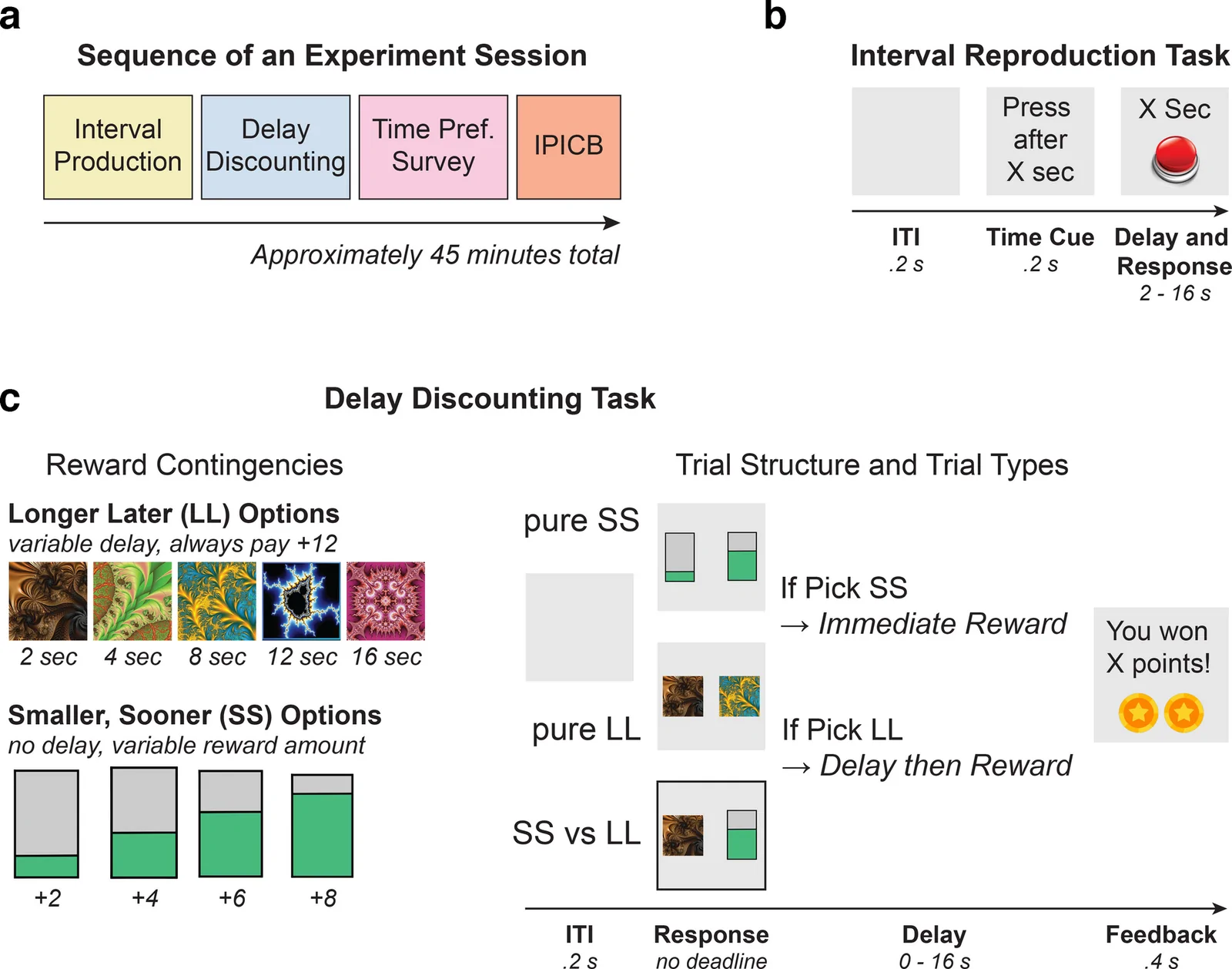
Experimental design. **a** Order of components within an experimental session. **b** Structure of the interval production task. After a brief training period, subjects began the main task. Trials begin by instructing the delay which needs to be produced. After the instructions, a red button appears to indicate the start of the interval. Participants press the SPACE bar to indicate when they think the interval has elapsed. **c** Structure of the delay discounting task. Participants complete two training blocks (20 trials each) to learn stimulus–reward associations, followed by a 20-min period total where they accumulate points. Trial types were presented in random order with equal frequency. Three trial types: pure SS trials (comparison between two SS options), pure LL trials (comparison between two LL options), and SS vs LL trials. The choice options were either colored bars, which represent smaller, sooner rewards (+ 2, + 4, + 6, + 8 points, delivery after 1 s, regardless of reward level) or fractals, which represent larger, later rewards (all + 12 points delivered after 2, 4, 8, 12, or 16 s). (Color figure online)

We tested our hypotheses by recruiting 226 (94 women) human participants to complete an interval production task, an experiential delay discounting task, and a temporal preference survey, and a survey about their timing-related behaviors outside of the laboratory (Fig. 1a). The mean ± *SD* age was 41 ± 13 years old. The experiment was hosted online using the *jsPsych* library (de Leeuw et al., 2023) and participants were recruited through *Prolific*.

### Establishing task comprehension and baseline behavior

#### Quantifying clock speed and precision

**Fig. 2:**
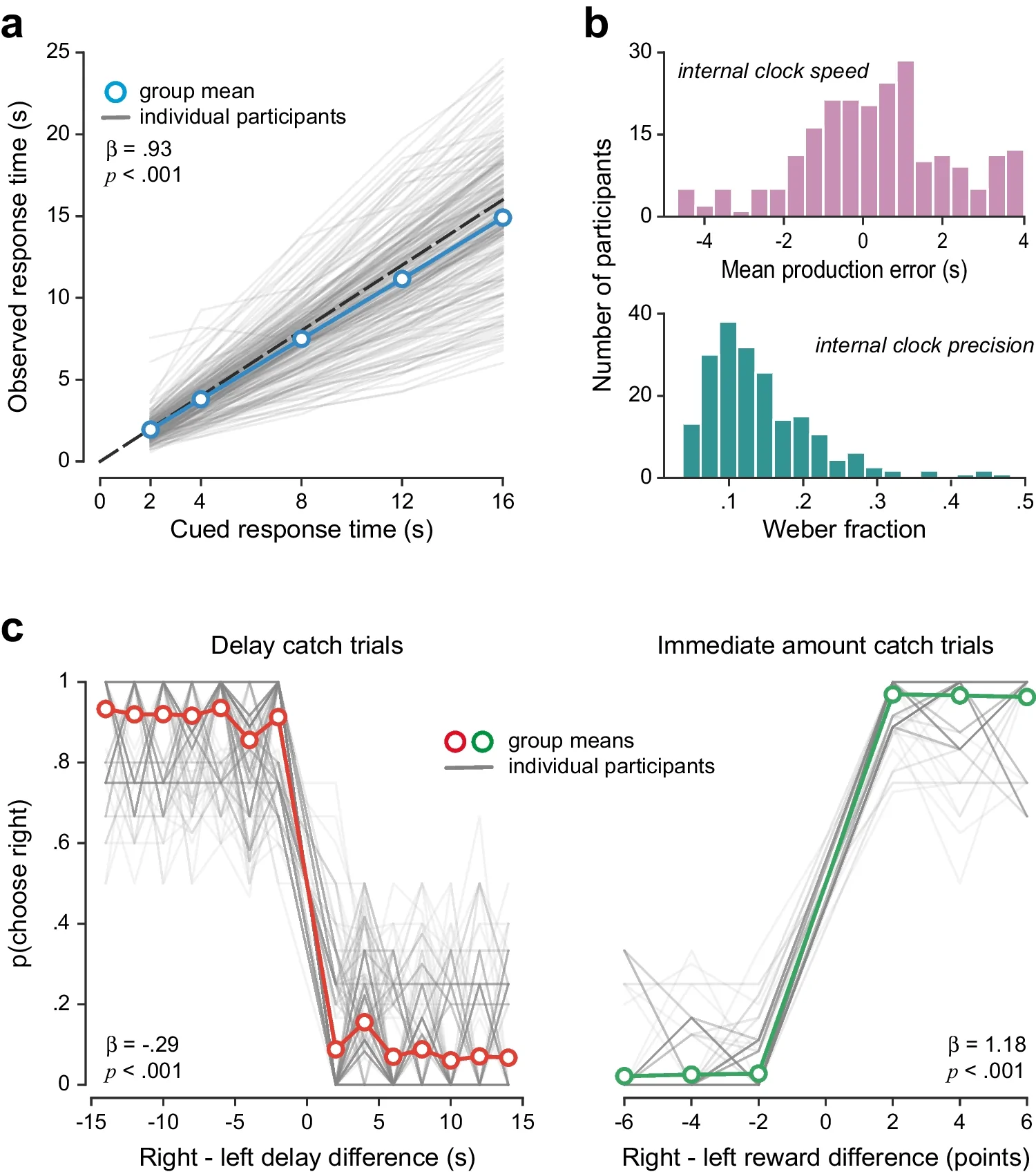
Behavioral performance during the interval production task and EDT catch trials. **a** Response times as a function of cued time during the interval production task. **b** Distribution of mean production errors (top) and Weber fractions (bottom) during the interval production task. **c** Psychometric functions during the “pure LL” and “pure SS” catch conditions during the EDT. (Color figure online)

Clock speed reflects systematic biases in time perception—whether an individual’s internal clock runs consistently faster or slower than objective time. We quantified clock speed as the difference between the time people were cued to wait before responding and their actual response. On average, people tended to underproduce the target interval (β = 0.93, *p* <.001, mixed-effects regression; Fig. 2a). We then assessed each subject separately; all subjects exhibited significant, positive beta weights (all *p* <.01), indicating that participant response times tracked the cued time. In other words, this indicates that participants understood the interval production task. However, we observed significant variability amongst individuals (Fig. 2b)—some people systematically press too early or late, consistent with a faster or slower internal clock, respectively.

We also quantified the precision of people’s internal clocks by calculating the Weber fraction of production times for each participant (see Methods; Fig. 2b). Earlier work established that variance in timing judgments linearly increases with interval duration, following Weber’s law (Gibbon, 1977). The Weber fraction normalizes for this relationship by dividing the standard deviation by the mean production time, providing a scale-invariant measure of temporal precision (Gibbon, 1977; Haigh et al., 2021; Wearden & Lejeune, 2008). We observed a median Weber fraction of 0.13, which is consistent with prior reports of human timing variability (Haigh et al., 2021). Our participant Weber fractions were right skewed, indicating that we also obtained sufficient variability in timing precision across our participant pool.

#### Quantifying task comprehension during the experiential discounting task

Following the interval discrimination task, participants completed an experiential delay discounting task (EDT) where they chose between smaller, sooner rewards and larger delayed rewards (Fig. 1c). We modelled our task after Kobayashi and Schultz’s (2008) seminal study on delay discounting in dopamine neurons. Figure 2c shows choice performance during the catch trials where participants choose only between either two options with the same reward amount but different delays (Fig. 2c, left) or between two options with the same delay but different reward amounts (Fig. 2c, right). We used mixed-effects logistic regression to assess behavior during these conditions, which revealed that participants tended to choose the shorter delay during delay catch trials and tended to choose the larger reward option during the immediate reward catch trials. Together, these results indicate that participants understood the stimulus-reward and stimulus-delay mappings and performed well when time and reward did not require a trade-off.

### Dissociating willingness to delay gratification in survey-based versus experiential discounting tasks

**Fig. 3:**
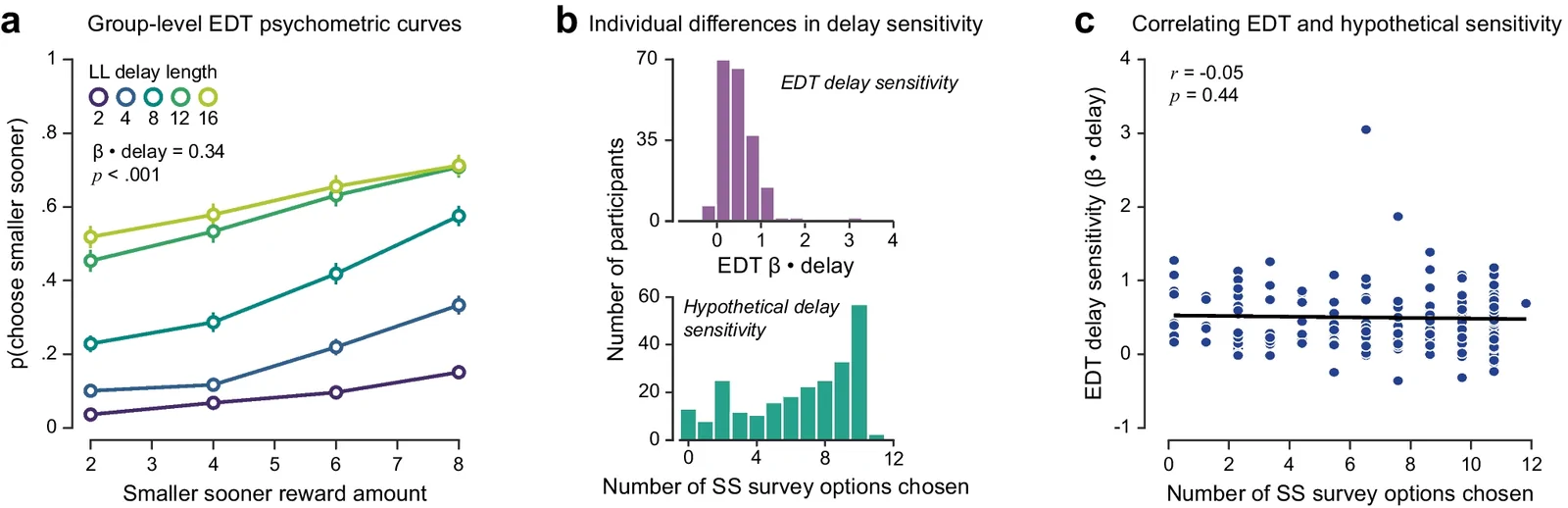
Delay sensitivity during the EDT and a hypothetical time preference survey.** a** Likelihood of choosing the smaller sooner (SS) option as a function of the SS amount and delay length of the longer, later option during SS versus LL trials in the EDT. **b** Individual differences in delay sensitivity during the EDT (top) and time preference survey (bottom). **c** Delay sensitivities based on hypothetical preferences and experience are not correlated with each other. (Color figure online)

Having established that participants understood the tasks, we next asked how they traded off delays and rewards. For the EDT, we used mixed-effects logistic regression to examine how our participants traded off rewards and delays when the two were pitted against each other during *SS vs. LL* trials. As a group, our participants were more likely to choose a smaller, sooner option as both the amount of that option increased (β = 0.37, *p* <.001, mixed-effects logistic regression) and as the length of the delay associated with the larger, later option increased (β =.34, *p* <.001; Fig. 3a). We did not observe a delay × amount interaction (β =  − 0.001, *p* = 0.58). To characterize individual differences in delay sensitivity in the EDT, we fit a logistic regression to each individual participant. As shown in Fig. 3b (top), we observed substantial individual differences—meaning that there was a sufficient range of delay sensitivities across our participant pool. We also measured delay sensitivity in a time preference survey, which measured how people trade off hypothetical rewards and delays across 12 choice scenarios (Bartels et al., 2023). The survey is scored as the sum of the number of smaller, sooner options chosen. We observed substantial individual differences in delay sensitivity in such hypothetical scenarios (Fig. 3b, bottom). Next, we asked whether people’s time preferences for experienced and hypothetical delays were correlated (Fig. 3c). They were not (*r* =  −.05, 90% CI: [−.16,.06], *p* =.44). To assess whether this null result reflected a meaningful absence of association rather than insufficient evidence, we conducted a two one-sided tests (TOST) procedure (Lakens, 2017) to test our preregistered equivalence bounds of ±.15. The observed correlation was significantly bounded from above (*p* =.001) but not from below (*p* =.066). We further probed the true *r* using a more lenient bound of ±.20, against which the correlation was statistically equivalent to zero (lower: *p* =.011; upper: *p* <.001). Together, these results suggest that any association between experienced and hypothetical time preferences is small, with the data ruling out effects larger than ±.20 in either direction.

### Dissociating willingness to delay gratification from behavioral measures of time perception

**Fig. 4:**
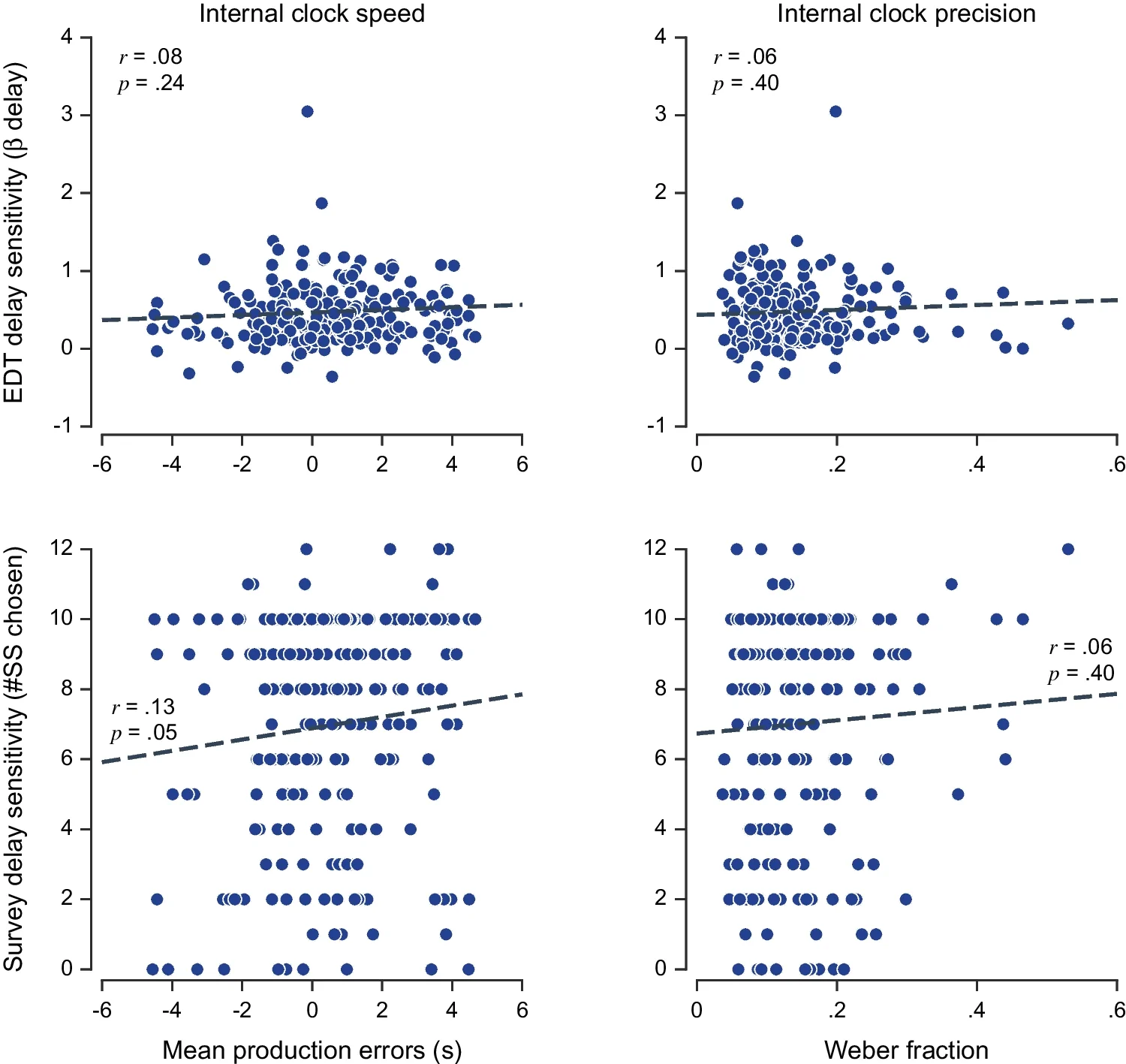
Willingness to delay gratification is unrelated to behavioral measures of time perception. The left column shows delay sensitivity during the EDT (top) and time preference survey (bottom) as a function of mean production error during the interval production task. The right column shows these measures as a function of internal clock precision, quantified as the Weber fraction during the interval production task

Next, we examined the relationship between willingness to delay gratification and time perception (Fig. 4). Using correlations, we found that our measures of clock speed and precision were unrelated to delay sensitivity measured in the EDT (production error: *r* =.08, 90% CI: [−.03,.19], *p* =.23; clock precision: *r* =.06, 90% CI: [−.05,.17], *p* =.37). For delay sensitivity measured through the temporal preference survey, clock precision was likewise uncorrelated (*r* =.06, 90% CI: [−.05,.17], *p* =.37), while production error trended towards a small, positive association (*r* =.13, 90% CI: [.02,.24], *p* =.05). Notably, the direction of this trend runs opposite the prediction of the internal clock speed hypothesis, under which faster clocks (more negative production errors) should be associated with greater impatience. Applying the same logic as the TOST procedure, all four 90% confidence intervals fall within ±.24, indicating that any true association between timing behavior and delay sensitivity, if it exists, is small in magnitude.

### Exploratory analysis

#### Relating time perception and delay sensitivity to behaviors outside of the laboratory

Although the goal of our experiment was to understand whether individual differences in time perception relate to differences in willingness to delay gratification, we were also interested in exploring how well discounting and time perception tasks predict behavior outside of the laboratory. Therefore, in addition to the behavioral tasks and time preference survey, participants also completed the newly developed Intertemporal Preferences in Choice Behavior (IPICB) scale (Krefeld-Schwalb et al., 2024), a self-report instrument that measures stated tendencies toward patient behavior across personal finance, health, and effort domains. We present the 20 items comprising the IPICB scale in Table 2.

**Fig. 5:**
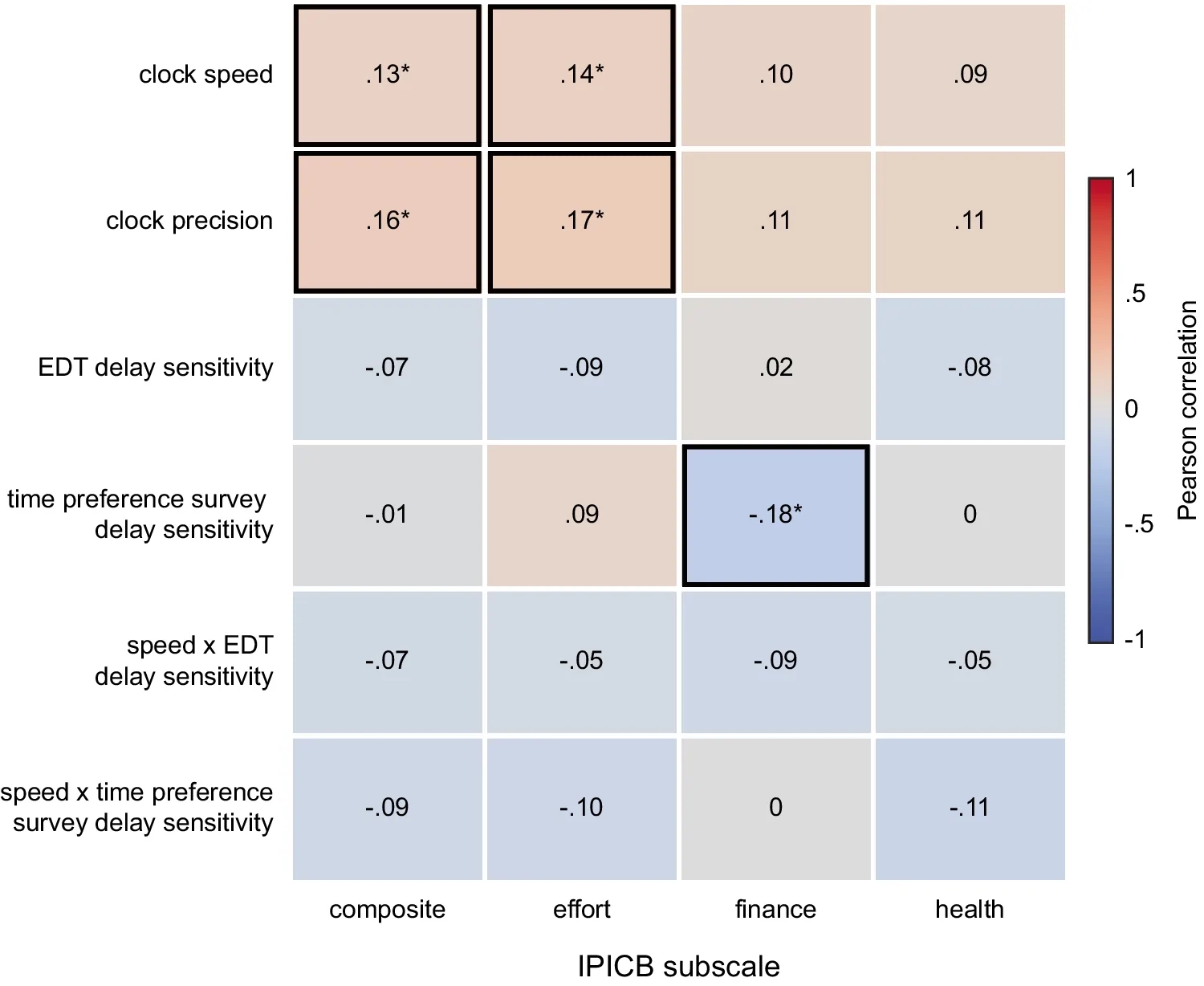
Relating timing behaviors in and out of the laboratory. Each box represents a pairwise Pearson correlation, the magnitude of which is indicated by the inset number. Bold-outlined boxes and asterisks indicate *p* <.05, uncorrected. (Color figure online)

We observed small but significant correlations between our laboratory measures of time perception and the overall scores on the IPICB (Fig. 5). We also observed a significant relationship between responses on our time preference survey and the financial IPICB subscale—which matches the time preference survey in that both asked questions about hypothetical financial decisions. However, the vast majority of the tested relationships were not significantly different from 0. Notably, the EDT was not significantly related to the IPICB composite score or any of its subscales, and the time preference survey related only to the finance subscale. Taken together, our results indicate the hypothetical and experiential measures of time preferences derive from cognitive processes which are not only unrelated to each other—but also largely unrelated to self-reported timing-related behaviors outside of the laboratory.

## Discussion

In this registered report we tested the central prediction of the “internal clock speed hypothesis” (Wittmann & Paulus, 2008)—that individual differences in the willingness to delay gratification derive from individual differences in time perception. We found no significant relationship between any measure of time perception and temporal discounting, measured through both an experiential discounting task (Kobayashi & Schultz, 2008) and a time preference survey (Bartels et al., 2023). Like others (Melanko et al., 2009; Patt et al., 2021; Reynolds et al., 2006; Smits et al., 2013; Steele et al., 2019), we observed no significant relationship between survey-based and experiential measures of delay-discounting behavior; our results extend this prior work by showing that neither measure relates to time perception or to timing-related behavior outside the laboratory. Together, our findings indicate that survey-based temporal discounting, experiential temporal discounting, and time perception derive from dissociable cognitive processes.

A critical aspect of interpreting our study is that our participants understood the tasks and surveys. An alternative explanation could have been that our results reflect a failure to learn the stimulus-delay/reward associations in our experiential discounting task. However, this was not the case: participants performed near ceiling during catch trials where they chose between options that differed only in their immediate reward amount or the delays associated with options that paid the same amount, respectively. Moreover, we observed clear effects of temporal discounting during the key trials where participants chose between options that yielded a smaller, sooner versus a larger, later reward. Similarly, we observed clear evidence that people understood the delay production task: All participants exhibited significant, positive relationships between their response time and the cued time. This indicates that rather than a failure of task comprehension, our results reflect true dissociations between survey and task-based measures of temporal discounting, these measures and time perception, and these measures and behaviors outside of the laboratory.

One candidate explanation for the dissociation between time perception and delay discounting is that these tasks make fundamentally different demands on temporal cognition. The interval production task requires participants to track the ongoing passage of time—an inherently present-tense process. Delay discounting, by contrast, requires participants to mentally project themselves into the future and evaluate a reward they have not yet experienced. Consistent with this, Shamosh et al. (2008) found that working memory capacity, which supports the active maintenance and manipulation of future-oriented representations, was negatively correlated with temporal discounting (*r* =  −.4). Future research could more precisely examine how delays and future rewards are represented in working memory (Kennerley & Wallis, 2009; Miller et al., 2018) and, critically, how neural representations of simulated time shape the process of valuation (Elston & Wallis, 2022, 2025).

A key aspect of our results is that laboratory measures of temporal discounting have limited ability to predict self-reported timing-related behaviors outside of the laboratory. Our results are consistent with recent work by Bartels et al. (2023), who also observed small relationships between survey-based measures of discounting and self-reported benchmarks of real-world timing behaviors. One interpretation of this disconnect is that experiential tasks like ours could be viewed as performance rather than preference measures: Because the task’s incentive structure parametrically determines an optimal response, participants who successfully learn the task contingencies may converge on similar behavior regardless of their underlying time preferences. However, we observed substantial individual differences in delay sensitivity during the EDT (Fig. 3b), even though participants clearly understood the task contingencies as evidenced by near-ceiling performance on catch trials (Fig. 2c). This pattern suggests that participants did not simply converge on a single optimal strategy, but rather expressed genuine variation in how they traded off time and reward within the task.

Survey-based measures of time-preference could, in principle, sidestep the performance-versus-preference concern by asking participants directly about their real-world inclinations. Yet our data suggest that these too have limited predictive validity: The time preference survey was unrelated to the IPICB composite and only correlated with the financial subscale, which closely matched the survey’s own hypothetical monetary framing (Fig. 5). Together, these findings suggest that current laboratory measures and time-preference surveys may not cleanly capture the cognitive processes engaged when people make intertemporal decisions in everyday life.

Foraging paradigms, such as patch-leaving (Blanchard & Hayden, 2015; Constantino & Daw, 2015; Hayden et al., 2011; Kendall & Wikenheiser, 2022), offer a promising alternative for two reasons. First, the cost of time is never explicitly presented to the forager but must be learned and continuously updated from experience. Second, and more importantly, foraging behavior is well described by the marginal value theorem (Charnov, 1976), which states that an optimal forager should leave a depleting resource patch when its instantaneous rate of return falls to the average rate available in the environment. Such decisions inherently require weighing the diminishing value of staying against the time cost of traveling to a new patch. This framework provides an established computational basis for understanding the cognitive and neural mechanisms underlying time–reward trade-offs. Moreover, organisms as diverse as humans, monkeys, seals, and even plants have been shown to behave according to the marginal value theorem (Agetsuma, 1998; Bendesky et al., 2011; McNickle & Cahill, 2009; Smith & Winterhalder, 1992; Thompson & Fedak, 2001). This generality suggests that the computations formalized by the marginal value theorem may be fundamental to the way organisms make decisions that trade off time and reward. Future research could therefore use foraging paradigms to not only obtain more ecologically valid measures of temporal decision-making, but also to leverage a mature theoretical framework to understand the cognitive processes and neural mechanisms that underlie them.

In sum, we tested the “internal clock speed hypothesis” (Wittmann & Paulus, 2008) and did not observe its central prediction of individual differences in time perception explaining individual differences in temporal discounting behavior. Like others (Melanko et al., 2009; Patt et al., 2021; Reynolds et al., 2006; Smits et al., 2013; Steele et al., 2019), we found no clear relationship between survey-based and experiential, task-based measures of delay-discounting behavior, or between these measures and time-related behaviors outside of the laboratory. We highlight foraging tasks as a promising future direction because they offer an intrinsically more naturalistic design and a mature computational framework, the marginal value theorem, for understanding the cognitive and neural mechanisms underlying decisions that trade off time and reward.

## Methods

### Ethical approval

This study was reviewed and approved by the Institutional Review Board at the University of Texas at Austin. All participants provided informed consent before beginning the experiment. Participants were informed that their participation was voluntary and that they could withdraw at any time without penalty. No personally identifiable information about participants was collected.

### Power analysis

We conducted an a priori power analysis to determine adequate sample size for detecting correlations between individual differences in time perception and delay discounting behavior. The most directly relevant prior research to our study comes from Kim and Zauberman’s (2009) study, which found that individuals who perceived hypothetical future delays as subjectively longer showed steeper discounting (*r* = 0.31). Based on the theoretical prediction that direct behavioral measures of timing and delay discounting should be at least as correlated as survey-based methods, we powered our study to detect correlations of *r* > 0.30.

We planned two correlational analyses: (1) discount rate with temporal discrimination thresholds (Weber fractions), and (2) discount rate with interval production accuracy. With Bonferroni correction for these two comparisons (*α* = 0.025), power calculations using Fisher’s *r-to-Z* transformation indicated that *N* = 133 participants will provide 90% power to detect *r* > 0.30.

However, in order to conduct the two one-sided test (TOST) equivalence testing procedure, we increased our sample size to 190. See the “Equivalence Testing” section and Eqs. 4–7 below for details. We also examined the correlation between temporal discrimination and interval production to explore whether these timing behaviors arise from a common underlying mechanism. We inadvertently recruited 226 participants and our sample size of N = 226 had > 80% power to detect a correlation of *r* > 0.30 between these measures.

### Open science practices

All raw, trial-level data, survey responses, Javascript code, and python analysis code are publicly available at the project’s Open Science Framework page: https://osf.io/6nkbz/overview?view_only=35230505817946e584614837f9259f8c.

### Participants

#### Recruitment

We recruited 226 participants through prolific.com, an online platform. We accepted any participant that resides in the United States, is fluent in English, has normal or corrected-to-normal vision, and was at least 18 years old at the time of study recruitment. The experiment took approximately 45 min to complete, and participants were compensated $9 for their time ($12/hour).

#### Exclusion criteria

We excluded participants on the basis of task performance, task engagement, and completion of the entire experiment. Our performance criteria was based on the “catch” trials of the delay discounting task—trials where participants choose between either two smaller, sooner options that varied in their reward amounts or two larger, later options that vary in their delay amounts. The best choice in the smaller, sooner catch trials is the one which pays a greater reward whereas the best choice in the larger, later catch trials is the one with the shorter delay (since all larger, later options pay + 12). Participants that scored less than 65% across these “catch” conditions were excluded from further analysis. Our final dataset reflects subjects who passed this criterion as well as completed all experimental components.

### Online behavioral testing

We programmed our experiment using the *jsPsych* JavaScript library (de Leeuw et al., 2023) and hosted the experiment online through cognition.run. Cognition.run is an online platform for hosting behavioral experiments programmed in *jsPsych* that can integrate with Prolific, the platform where we will recruit our participants. Prior work (Anwyl-Irvine et al., 2021; Bridges et al., 2020) demonstrated that *jsPsych* maintains high timing accuracy when running experiments online and is widely and routinely used for reaction time studies.

### Behavioral tasks

#### Interval production task

The interval production task measured participants’ ability to accurately produce specific time intervals using their internal clock. On each trial, participants were first presented with a visual cue indicating the target duration they need to produce (e.g., “4 s”). A red button then appeared on the screen, signaling that the timing period had begun. Participants pressed the SPACE bar when they believed the cued duration had elapsed. The task required participants to rely solely on their internal sense of time, as no external timing cues were provided during the production period.

The task began with brief instructions explaining the production procedure. Subjects were then given a set of five practice trials, one for each duration of 2, 4, 8, 12, and 16 s. We used these delays because they correspond to the ones used in our delay discounting task, ensuring that our measure of time perception occurred within the same timescale as our decision task. Participants received no feedback about their responses. After the practice block, participants proceeded to the experimental block, where each target duration was presented 10 times in a randomized order.

#### Delay discounting task

We modelled our delay discounting task after the one used by Kobayashi and Schultz (2008), which measures willingness to wait for larger rewards by presenting choices between smaller, sooner rewards and larger delayed rewards. This experiential paradigm required participants to experience the delays associated with their choices, as compared to designs where people imagine decisions between different reward amounts and delays. The task consisted of three sequential blocks designed to familiarize participants with the reward contingencies before measuring their temporal preferences. The order of the training blocks was counterbalanced across participants.

##### Larger, later training block

This block of 20 trials was meant to teach participants the delay lengths associated with each of the “larger, later” (LL) options. On each trial, participants choose between different LL options, each represented by a distinct fractal that corresponds to a specific delay period (2, 4, 8, 12, or 16 s). This block allowed participants to experience and learn the delay contingencies associated with each fractal without the influence of competing immediate rewards. Participants experienced the delay associated with their choice prior to receiving their reward. All LL options provided the same fixed reward of + 12 points, so the optimal strategy was to choose the option with the shortest delay.

##### Smaller, sooner training block

This block of 12 trials was meant to teach participants the reward values associated with each of the “smaller, sooner” (SS) options. On each trial, participants chose between different SS options, each represented by colored bars (+ 2, + 4, + 6, + 8). This block allowed participants to learn the reward contingencies associated with each colored bar without the influence of competing delayed rewards. The height of the fill within the bar indicated the point value. All SS options were rewarded after a fixed 1-s, delay and participants received the number of points associated with their decisions. The optimal strategy was to choose the option with the highest point value.

##### Experimental blocks

The experimental block lasted 20 min, during which participants were instructed to collect as many points (coins) as possible. The experimental block was composed of “mini-blocks” with short, self-paced breaks between mini-blocks. Each mini-block was 72 trials—one full repetition of all choice conditions. Participants continuously completed these mini-blocks until 20 min have elapsed. Participants were only shown the time remaining during the breaks between the mini-blocks and the clock only ticked down during task performance (i.e., the self-paced pauses were not included in the 20 min).

These blocks included three trial types in randomized order: “Pure SS” trials presented two smaller, sooner options with different reward amounts (identical to the SS training block); “Pure LL” trials presented two larger, later options with equivalent + 12 point rewards but different delay periods (identical to the LL training block). “SS vs LL” trials presented one smaller, sooner option against one larger, later option, directly measuring temporal discounting behavior. The pure SS and pure LL trials served as “catch” trials to ensure task comprehension throughout the session.

When participants selected a smaller, sooner option, they received those points after a 1-s delay and proceeded to the next trial. When participants selected a larger, later option, they had to wait through the delay associated with their choice before receiving the + 12 points and continuing. This experiential component ensured that participants’ choices have real temporal consequences. Participants received feedback on their choices in the form of points earned and their total accumulated points were displayed throughout the session.

#### Control conditions during experimental blocks

We used two types of catch trials to ensure that participants understood the rewards and delays associated with the choice options. The “pure LL” trials were choices between two delayed options that both paid + 12 such that the best choice is the shorter one. Similarly, “pure SS” trials involve choices between two non-delayed options that pay different numbers of coins such that the one with greater reward is better. These control conditions were important because they ensure that our results cannot be explained as participants failing to understand the task and stimuli.

#### Reward rate considerations

Our task created meaningful trade-offs that prevent simple heuristics, as neither “always SS” nor “always LL” strategies yield optimal reward rates. With SS options requiring 3.1-s total (1-s delay + 2-s feedback + 0.1-s between trials) and LL options requiring their delay plus 2.1-s intertrial interval (ITI), reward rates vary systematically: the shortest LL option (2-s, + 12 points: 2.93 pts/s) exceeds lower SS options (+ 2 SS: 0.65 pts/s; + 4 SS: 1.29 pts/s) but competes with higher SS options (+ 6 SS: 1.94 pts/s; + 8 SS: 2.58 pts/s). Since reward rates fundamentally depend on subjective time experience, participants with different internal clock speeds could conceivably make systematically different choices, allowing us to measure how temporal processing influences decisions that trade off time and reward.

#### Time preference survey

We used the time preference survey reported in Bartels et al. (2023) to measure people’s time preferences during hypothetical, non-experiential decisions. Table 1 shows the choices participants were asked to make.

**Table 1:** 12-item battery of intertemporal choices from Bartels et al. (2023)

Smaller, sooner options	Larger, later option
$504 now	$524 in 1 month
$600 now	$611 in 1 month
$777 now	$791 in 1 month
$1064 now	$1153 in 1 month
$816 now	$5440 in 1 year
$213 now	$281 in 2 years
$816 in 6 months	$860 in 9 months
$816 in 6 months	$1028 in 1 year
$400 in 6 months	$440 in 1 1/2 years
$840 in 6 months	$10125 in 2 1/2 years
$791 now	$771 in 1 month
$621 in 6 months	$670 in 6 months

The final two items were attentional checks. The item order was randomly presented to participants

#### Intertemporal Preferences in Choice Behavior (IPICB) scale

We collected responses on the Intertemporal Preferences in Choice Behavior (IPICB) scale (Krefeld-Schwalb et al., 2024), which measures how people make time-value decisions across personal finance, health, and effort domains (Table 2).

**Table 2:** 20-item battery of the intertemporal preferences in choice behavior scale

Personal Finance subscale	
1	I would start saving for my retirement today, instead of spending more of my money while I’m young
2	If I bought new clothes over the next few months, I would buy high-quality clothing instead of cheaper clothing that I would need to replace sooner
3	If I had bills to pay today, I would pay them immediately instead of letting them pile up
4	If my credit card bill arrives at the end of the month, I would pay it in full instead of only paying a part of it
5	If my paycheck arrived next month, I would deposit some of it in a savings account instead of depositing everything into my checking account
Health subscale	
1	Before I go to bed tonight, I would brush my teeth and floss instead of only brushing my teeth
2	If I had a routine dental check-up scheduled for today, I would go to the checkup, even if I didn’t have insurance coverage, instead of canceling the checkup
3	If I was feeling chest pain today, I would go to the doctor today instead of waiting for it to get worse
4	If I went out for lunch next week, I would go to a restaurant which is a 10-min walk away because it offers healthier food (reduced sodium, sugar, and fat), instead of a restaurant that is only a 5-min walk away that offers less-healthy food
5	If my dentist offered me a special treatment to prevent tooth decay that my insurance doesn’t cover today, I would do it instead of saving the money
6	Today, I would exercise after work instead of just hanging out
Effort subscale	
1	I would do the dishes today instead of letting them pile up and having more to clean later
2	If I had a performance evaluation (at work or at school, etc.) a few months from now, I would start working on my skills today instead of shortly before the evaluation
3	If I had an exam next week, I would stay home the night before the exam instead of going out with friends
4	If I hosted a dinner party next weekend, I would clean up as soon as my guests leave, instead of cleaning up the next day
5	If I received a letter informing me that I need to send in additional documents regarding my taxes in a few months, I would prepare the documents today instead of postponing it to tomorrow
6	If I received a new task from my employer next week, I would start immediately instead of postponing it to the next day
7	If something in my house gets slightly damaged, I would fix it today instead of waiting for it to fully break
8	With my free time next month, I would work on learning and developing my skills instead of spending it on leisure time
9	If my kitchen cabinets need to be cleaned up, I would do it today instead of postponing it to the next day

Each item is scored with a Likert scale from 1 to 5, gathering the likelihood of respondents to do the behavior (1 = *very unlikely*, 2 = *unlikely*, 3 = *unsure*, 4 = *likely*, 5 = *very likely*)

### Data analysis

#### Assessing interval production behavior

We analyzed interval production performance by calculating production accuracy for each participant across all trials in the experimental block. For each trial, we computed the production error as the difference between the participant’s actual production time and the target duration:

1 $$Production\, Error=Actual \,Production \,Time-Target\, Duration$$

We then calculated each participant’s mean production error across all trials as well as their mean production error for each of the four target durations (2, 4, 8, 12, and 16 s). The overall mean production error was our primary measure of internal clock speed from the interval production task.

We also computed the Weber fraction for each interval and pooled them to derive a single precision metric:

2 $$Weber \,Fraction=Standard\, Deviation \,of \,Production\, Times/Mean\, Production \,Time.$$

#### Logistic regression to assess delay discounting

We analyzed delay discounting behavior using logistic regressions fitted to each participant’s choices during the experimental block, focusing specifically on the SS vs. LL trials where participants choose between smaller, sooner and larger, later options. We modeled the probability of choosing the smaller, sooner option as a function of the more immediate reward amount, the delay length, and their interaction:

3 $$p\left(choose\, SS\right)=\sigma \left({\beta}_{1}\times SS\_amount+{\beta}_{2}\times delay\_length+{\beta}_{3}\times SS\_amount\times delay\_length+{\beta}_{0}\right),$$

where *σ* represents the sigmoid function, *β₁* captures sensitivity to SS reward magnitude, *β₂* captures sensitivity to delay length (a key measure of delay discounting), *β₃* captures the interaction between reward amount and delay, and *β₀* represents the intercept. Our key analyses focus on *β₂*.

#### Equivalence testing

To strengthen inference from null results, we conducted equivalence testing using the two one-sided tests (TOST) procedure for correlations (Lakens, 2017). We preregistered equivalence bounds at *r* =  ± 0.15.

The TOST procedure for correlations uses Fisher’s *r*-to-*Z* transformation to conduct two one-sided tests: one testing whether the observed correlation is statistically smaller than the upper equivalence bound (*rU* = 0.15), and another testing whether it is statistically larger than the lower equivalence bound (*rL* =  − 0.15). For each test, we calculated the *Z*-transformed correlation values as:

4 $$ZL=\left[LN\left(\left(1+r\right)/\left(1-r\right)\right)/2-LN\left(\left(1+rL\right)/\left(1-rL\right)\right)/2\right]/\left(1/\surd \left(N-3\right)\right)$$

5 $$ZU=\left[LN\left(\left(1+r\right)/\left(1-r\right)\right)/2-LN\left(\left(1+rU\right)/\left(1-rU\right)\right)/2\right]/\left(1/\surd \left(N-3\right)\right),$$

where *r* is the observed correlation, *rL* and rU are the lower and upper equivalence bounds, and *N* is the sample size.

To determine the required sample size, we conducted an a priori power analysis following Lakens’s (2017) method. First, we converted our equivalence bound (*r* =  ± 0.15) to a standardized effect size (Cohen’s *d*) using the following equation:

6 $$d=2r/\surd (1-{r}^{2}),$$

where *r* is the equivalence bound and d is Cohen’s *d*. This yielded *d* = 0.303.

Next, we applied the power equation for equivalence tests detailed in Lakens (2017):

7 $$n=2{\left(z\alpha + z\beta /2\right)}^{2}/{\Delta }^{2},$$

where *Δ* is the standardized equivalence bound in Cohen’s *d* units (0.3 for *r* =  ±.15), *zα* is the critical value for the one-sided alpha level (.05), which equals 1.645 in our case. β corresponds to 1-minus-the desired power level. *zβ/2* is the critical value for half the beta level. By this procedure, we needed at least 187 participants to achieve 80% power.

## Data Availability

Raw, trial-level data collected from this study are publicly available at our Open Science Framework page, which can be accessed here: https://osf.io/6nkbz/overview?view_only=35230505817946e584614837f9259f8c.

## Code availability

The Javascript code used to implement this experiment and the corresponding analysis code are publicly available at the following Open Science Framework page: https://osf.io/6nkbz/overview?view_only=35230505817946e584614837f9259f8c.
